# Clinical application of a body area network-based smart bracelet for pre-hospital trauma care

**DOI:** 10.3389/fmed.2023.1190125

**Published:** 2023-08-01

**Authors:** Wei Han, Jin-Yang Yuan, Rui Li, Le Yang, Jia-Qin Fang, Hao-Jun Fan, Shi-Ke Hou

**Affiliations:** ^1^Institute of Disaster and Emergency Medicine, Tianjin University, Tianjin, China; ^2^Emergency Department of Shenzhen University General Hospital, Shenzhen, Guangdong, China; ^3^School of Microelectronics, South China University of Technology, Guangzhou, Guangdong, China

**Keywords:** wearable electronic devices, body area network, pre-hospital emergency care, trauma, clinical application

## Abstract

**Objective:**

This study aims to explore the efficiency and effectiveness of a body area network-based smart bracelet for trauma care prior to hospitalization.

**Methods:**

To test the efficacy of the bracelet, an observational cohort study was conducted on the clinical data of 140 trauma patients pre-admission to the hospital. This study was divided into an experimental group receiving smart bracelets and a control group receiving conventional treatment. Both groups were randomized using a random number table. The primary variables of this study were as follows: time to first administration of life-saving intervention, time to first administration of blood transfusion, time to first administration of hemostatic drugs, and mortality rates within 24 h and 28 days post-admission to the hospital. The secondary outcomes included the amount of time before trauma team activation and the overall length of patient stay in the emergency room.

**Results:**

The measurement results for both the emergency smart bracelet as well as traditional equipment showed high levels of consistency and accuracy. In terms of pre-hospital emergency life-saving intervention, there was no significant statistical difference in the mortality rates between both groups within 224 h post-admission to the hospital or after 28-days of treatment in the emergency department. Furthermore, the treatment efficiency for the group of patients wearing smart bracelets was significantly better than that of the control group with regard to both the primary and secondary outcomes of this study. These results indicate that this smart bracelet has the potential to improve the efficiency and effectiveness of trauma care and treatment.

**Conclusion:**

A body area network-based smart bracelet combined with remote 5G technology can assist the administration of emergency care to trauma patients prior to hospital admission, shorten the timeframe in which life-saving interventions are initiated, and allow for a quick trauma team response as well as increased efficiency upon administration of emergency care.

## 1. Introduction

A common term called the “golden hour,” which is based on the “trauma death curve” theory refers to an approximately 60-min window following a severe injury in which effective treatment is needed to reduce morbidity and mortality rates (1). In complex or difficult to reach areas, traditional emergency response systems may struggle to arrive at the scene of an accident in a timely manner, leading to missed opportunities for prompt care to be administered. In such situations, a device with more portability and effectiveness is needed to provide life support on site during transport to the hospital (2). This is especially crucial for large-scale emergencies in which a large number of patients require treatment within a short period of time or when there is potential for the limited availability of emergency response because the number of patients is higher than usual. As a result, additional methods of professional management and communication were needed during these events (3). Therefore, the development of a more efficient trauma care system pre-hospitalization was of great importance.

Wireless body area network (WBAN) is an emerging technology that allows for local area network communication while consuming low quantities of energy. Remote life-sign monitoring systems developed based on WBAN technology have been shown to significantly increase the data transmission rate compared to traditional healthcare systems (4). In traditional healthcare systems, information for most patients is collected and transmitted *via* wired methods, which lack flexibility and limit the users’ normal range of activities. WBAN technology can automatically collect and record physiological signals from the patient in different environments, such as home, office, or a hospital, without affecting normal activities. Various physiological parameters can be transmitted to hospitals or servers, promoting a more efficient and timely treatment. Furthermore, sensor nodes can be used to monitor the sudden onset of conditions in the patient and promptly notify hospitals and family members to provide timely treatment.

Vital signs such as blood pressure, heart rate, body temperature, and blood oxygen saturation are external readouts of various physiological activities in the human body and are basic indicators for judging whether the body is healthy. When abnormalities occur, vital signs show different degrees of change, corresponding to dynamic changes caused by disease occurrence, development, and resolution. Therefore, real-time monitoring and recording of human vital signs provide an important scientific basis for clinical diagnosis and timely treatment of patients and to ensure correct guidance is given to the nursing staff caring for patients. At present, conventional monitoring methods often require patients to stay still for a few seconds. Furthermore, medical staff is required to bring monitoring equipment to the patient’s bedside in order to measure and record specific data for each patient, which is quite inefficient. WBAN technology enables intelligent monitoring through distributed sensor nodes, collecting vital sign information from the human body in real-time and transmitting them online to hospital servers (5–9). This technology is particularly useful when needed in operating rooms, intensive care units (ICU), and other hospital wards (6, 10). However, to date, there have only been a few studies on applying this technology for use in emergency medical services (4).

Wearable devices can integrate various biosensors to monitor and record physiological information such as blood pressure, pulse, blood oxygen saturation, respiratory rate, body temperature, electrocardiogram data, or electromyogram data through attachment to the body. These devices have excellent mobility (7, 8) and use body area network technologies in addition to other new technologies, such as remote 5G interaction, to provide a remote, real-time monitoring solution for pre-hospitalized patients, thus informing both diagnosis and treatment in emergency care (8, 9). This study aimed to explore the impact of a multi-parameter integrated life-monitoring smart bracelet based on BAN technology for efficient and effective emergency treatment of patients prior to hospitalization. Our findings provided evidence for the development of wearable monitoring devices and remote emergency medical technology based on WBAN, as well as for improving the quality of trauma treatment for patients before hospitalization.

## 2. Materials and methods

### 2.1. General information

**Figure 1:**
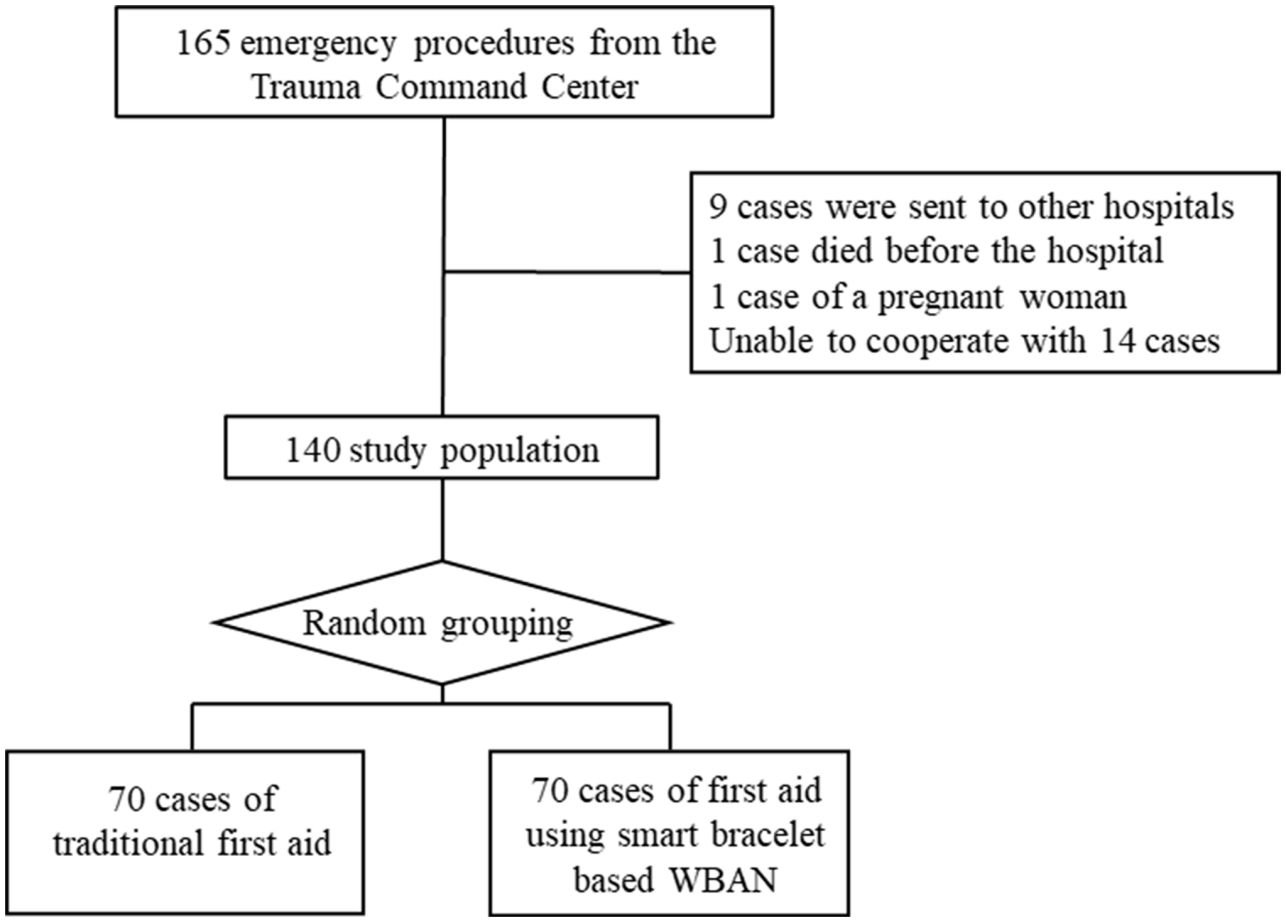
The study flow chart.

Clinical data from 140 pre-hospitalized trauma patients who were admitted to Shenzhen University General Hospital between June 10, 2022, and January 31, 2023, were analyzed in this observational cohort study. Inclusion criteria were: (1) trauma patients who were transported by the Shenzhen University General Hospital 120 Center and received treatment in the emergency department; (2) aged 18 to 80 years; (3) those who provided informed consent. Exclusion criteria were the following: (1) patients with mental disorders or unwilling to cooperate; (2) pregnant women; (3) patients who were confirmed dead after their initial assessments. The inclusion and exclusion process for this study is further detailed in Figure 1.

The sample size was calculated by GPower 3.1.9.7 software. The statistical method of t-test for two independent samples was applied; effect size (d) was set to 0.5, power of the test (1-β) was 0.8, and significance level (α) was set to 0.05. Each group required at least 64 participants. Therefore, 70 participants were included in each of the two groups (control and test groups) in this study; the experimental group consisted of 70 pre-hospitalized trauma patients who were treated using smart bracelets containing body area network technology, while the control group consisted of 70 pre-hospitalized trauma patients who were treated using traditional methods. Before data collection, grouping was completed by a random method. Specifically, samples were numbered 1–140 in advance, and each sample was randomly assigned a random three-digit number using the random number table. Then, the samples are sorted based on their three-digit number. According to the sorting results, the top 70 samples are divided into a control group, while the rest of them are divided into a test group. The experimenter decides whether to use the test equipment according to the group of patients who are presented sequentially.

This study was approved by the ethics committee of Shenzhen University General Hospital (Ethics Approval No. SUGHKYLL2022061001). It was conducted in strict compliance with relevant regulations and ethical guidelines. Informed consent was obtained from all patients or their family members. Obtaining written informed consent at the pre-hospital scene can be challenging and may hinder emergency rescue work. Therefore, we only obtained verbal informed consent from patients or family members at the scene, with written informed consent signed at the hospital. In cases where patients could not provide verbal consent and had no family members present, such as those who are unconscious, the patients still wore bracelets, and written informed consent was given by the family members at the hospital.

### 2.2. Experimental equipment

**Figure 2:**
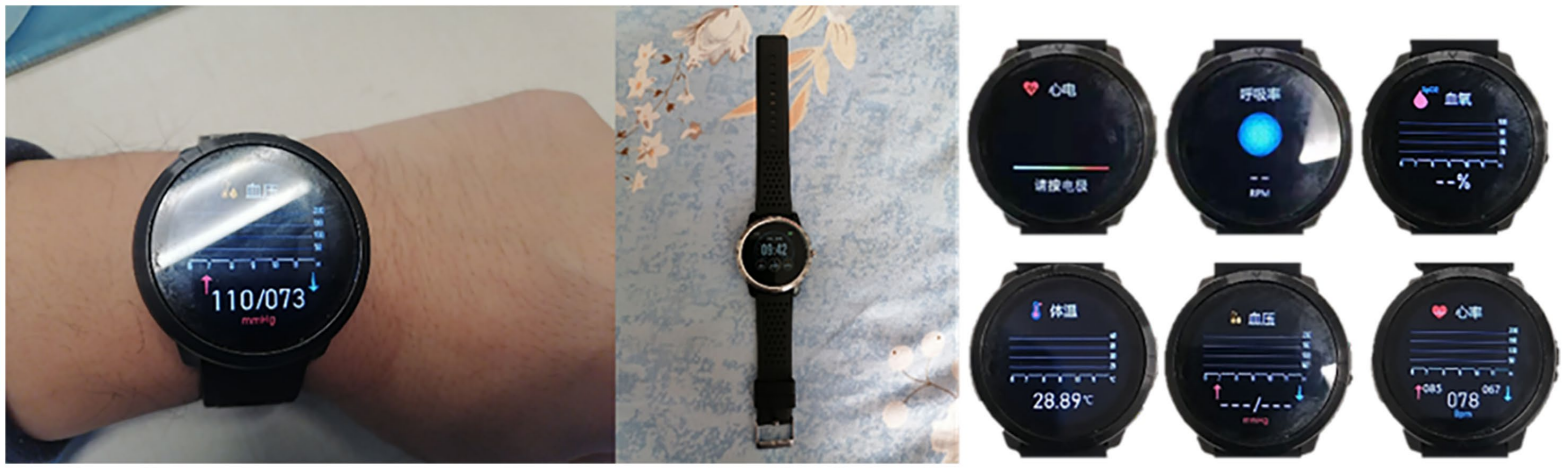
Emergency smart bracelet.

The experimental device used for this study was a multi-parameter integrated life-monitoring smart bracelet based on BAN technology, which was independently developed by our team, as shown in Figure 2. This smart bracelet can simultaneously monitor blood pressure, heart rate, blood oxygen saturation, body temperature, and respiratory rate, and perform single-lead electrocardiography.

**Figure 3:**
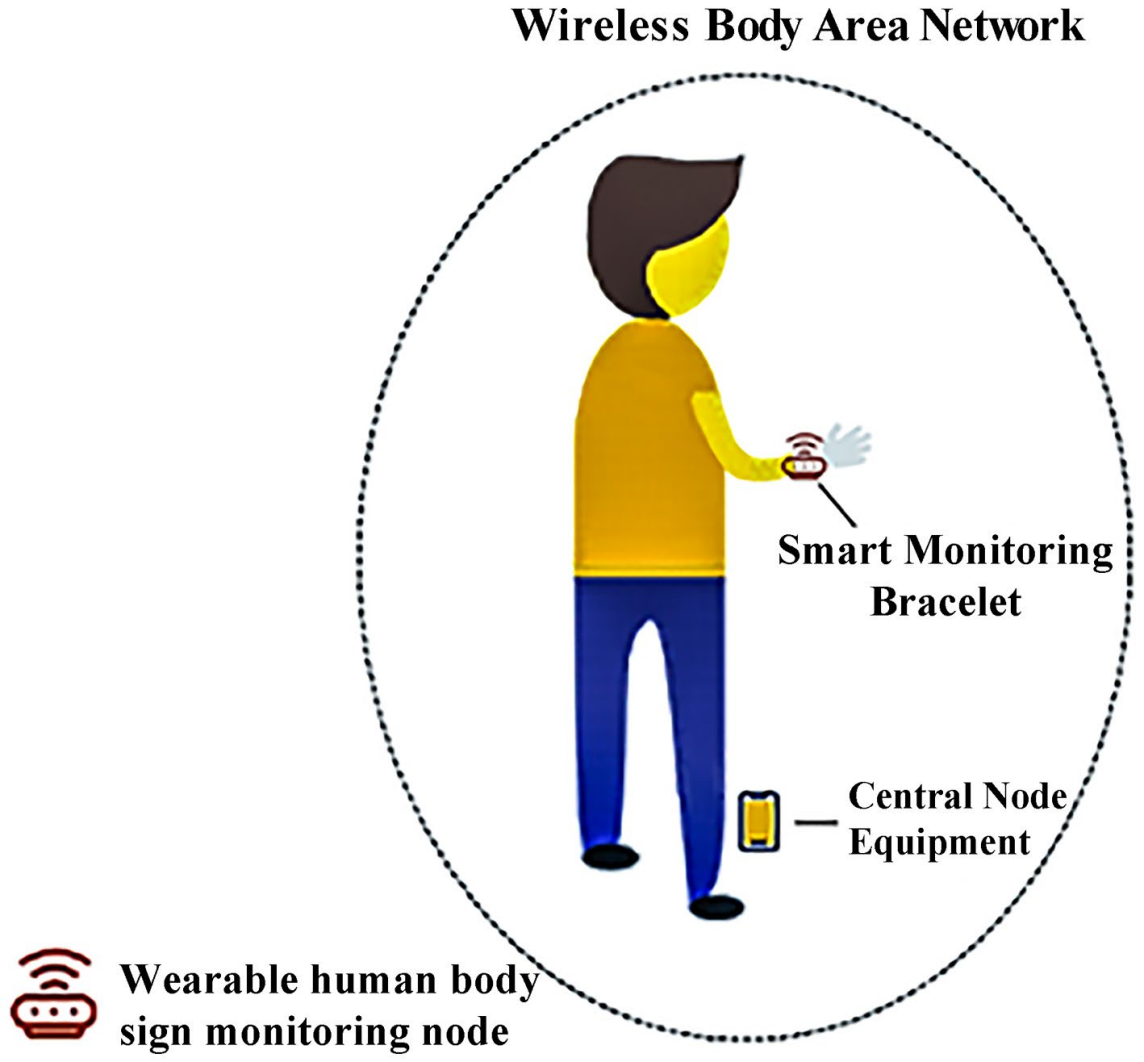
Wireless body area network.

The sensor component used in the bracelet was based on a Nordic52832 control chip, which includes an oxygen chip (TI high-performance analog front end AFE4404 + 2*Osram2703 PD + Osram three-in-one LED), temperature sensor (CT1711 array), electrocardiogram chip (Ti chip 129X), photoelectric chip (Ti AFE4404 + double Osram2703), heart rate chip (Yiguang PD70), and a gravity sensor (Rome KXTJ3-1057). The installed communication module uses a low-power 4.2BLE Bluetooth module, which requires the central node device (Figure 3) to be compatible with Android 4.4 or higher, IOS 8.0 or higher, as well as support Bluetooth 4.0. The hardware performance parameters were as follows: (1) the bracelet contains a memory of 512 KB (Flash 64 M); (2) the screen display was approximately 1.3” IPS 240×240; (3) the battery capacity was 240mAh, which allowed for 15-day standby periods or 5–7 days of full-time monitoring; (4) the bracelet supported physical buttons; (5) a built-in motor for vibration reminders; (6) it uses magnetic charging interface; (7) the waterproof rating for the body of the bracelet easily met IP67 standards. The bracelet can collect patient vital signs (blood pressure, blood oxygen saturation, heart rate, respiratory rate, temperature) in real-time. After wearing and completing the first-time measurement, we obtained blood pressure, blood oxygen saturation, and respiratory rate measurements at a frequency of 20 Hz, and obtained heart rate and temperature at 60 Hz.

The equipment used in this study included traditional life sign monitoring equipment that is commonly employed in the hospital prior to admission, which included: electronic blood pressure cuffs (Yuwell YE680A), pulse oximeters (Edan H100B), infrared thermometers (Fudakang KM-WD01), 12-lead electrocardiograph machines (Edan SE1201), as well as a vehicle-mounted defibrillator monitor (Mindray BeneHeart D6). The respiratory rate of patients was measured prior to hospital admission through visual estimation or stethoscope.

### 2.3. Emergency rescue methods

The control group underwent standard emergency rescue. Upon receiving a trauma emergency rescue task from the center, staff from the emergency department performed pre-admission vital sign monitoring *via* traditional emergency equipment upon arrival at the injury scene. The patients’ medical histories were obtained, their vital signs were measured, and a physical examination was performed to assess initial patient conditions. On-site treatment was provided as needed, and it included: the opening of patient’s airways, establishing venous access, oxygen supplementation, as well as other interventions such as tracheal intubation, cricothyroidotomy, needle decompression, and fluid replacement. After the staff completed on-site treatment, patients were transported by ambulance to the nearest trauma center. In the ambulance, patients’ cardiac statuses were monitored using a vehicle-mounted electrocardiogram measuring heart rate, blood pressure, pulse, oxygen saturation, and respiratory rate. The trauma team was activated upon arrival at the hospital, and a treatment plan was prepared based on the patients’ condition *via* phone or direct network communication.

For the experimental group, a smart wristband based on BAN technology combined with traditional equipment was applied for vital sign monitoring. Furthermore, remote communication was conducted through 5G internet technology before and after hospitalization. The study researchers did not interfere in any routine emergency rescue procedures. After obtaining consent from the patients or their family members upon arrival at the scene, the wristband was put on to monitor the patients’ blood pressure, heart rate, blood oxygen saturation, respiratory rate, and body temperature. The wristband data was connected to the BAN of the central node device and synchronized in real-time to the emergency physicians’ terminal in the hospital *via* 5G signaling. The active emergency physician in the hospital guided patient treatment using an online screen video according to the patients’ condition. Furthermore, the emergency department doctor activated the trauma team while preparing a patient rescue plan based on their conditions.

### 2.4. Variable definitions

The amount of time needed to administer the patient’s first rescue intervention, the amount of time needed to start a blood transfusion, the amount of time until the first use of hemostatic drugs, as well as 24-h and 28-day mortality rates were the primary variables. The secondary variables included the time necessary to activate the trauma team as well as the length of stay in the emergency department.

The evaluation indicators for treatment efficiency included: (1) rescue intervention measures, such as endotracheal intubation, cricothyrotomy, needle decompression, fluid replacement, use of hemostatic drugs (tranexamic acid), and blood transfusion; (2) the amount of time needed to begin patients’ first rescue intervention after their initial encounter with medical personnel prior to hospitalization; (3) amount of time necessary to begin a blood transfusion after emergency department admission; (4) amount of time between emergency department admission and the patients’ first use of hemostatic agents; (5) amount of time between the initial encounter with medical personnel prior to hospitalization to activation of the in-hospital trauma team.

The evaluation indicators of treatment effectiveness included: (1) mortality within 24-h of hospitalization, defined as the proportion of patients who died for any reason within 24-h after admission to the emergency department in each group; (2) 28-day mortality, defined as the proportion of patients who died for any reason within 28-days after injury in each group; (3) total time spent in the emergency department from admission to discharge.

### 2.5. Data collection

Data collection was performed by the research team prior to and after hospitalization. Pre-hospitalization data was collected in the ambulance and included vital signs measured by the smart bracelet and conventional equipment (blood pressure, heart rate, oxygen saturation, respiratory rate, and temperature), the site and type of injury, the injury severity score (ISS), time of arrival at the scene, time life-saving interventions were initiated, the time of trauma team contact at the hospital, the time of ambulance entry, as well as the time of emergency department arrival. In-hospital data was collected by a thorough review of patient records and nursing documents and included blood transfusion times, the use of hemostatic drugs, as well as the time patients left the operating room. The research team did not participate in clinical decision-making or treatment during these processes.

### 2.6. Statistical analyses

Statistical analyses were performed using SPSS Statistics 27.0 software (International Business Machines Corporation, United States). Continuous variables were expressed as mean ± standard deviation if normally distributed or as median values (interquartile range) if not normally distributed and were compared using student’s t-test or Mann–Whitney U test as appropriate. Categorical variables were expressed as frequencies or percentages and compared using a chi-square test or Fisher’s exact test. Kendall’s tau-b test was used to assess the consistency of the first measurement results from each type of equipment. *p* < 0.05 represented statistical significance.

## 3. Results

### 3.1. Characteristics of the study population

There were no statistically significant differences (*p* > 0.05) in the general characteristics between the test and control groups. The main mechanism of trauma in both groups was car accident injury and falling injury, without a statistical difference between the two groups (*p* > 0.05). The most common trauma sites in both the control group (28.57%) and test group (35.71%) were the limbs. The control group consisted of 8 patients with head and neck trauma (11.43%), 13 patients with thoracic trauma (18.57%), 18 patients with abdominal trauma (25.71%), and 6 patients with trauma in multiple areas (8.57%). The test group consisted of 5 patients with head and neck trauma (7.14%), 12 patients with thoracic trauma (17.14%), 15 patients with abdominal trauma (21.43%), and 8 patients with trauma in multiple areas (11.43%). There was no significant statistical difference (*p* > 0.05) in the main trauma sites between the two groups. There were 30 patients (42.86%) in the control group and 28 patients (40.00%) in the experimental group who had severe trauma (ISS > 16 points); there was no significant statistical difference (p > 0.05) in the proportion of patients with severe trauma between the two groups (Table 1).

**Table 1:** Comparison of general characteristics between groups.

Project	Control group	Test group	*p*
*N*	70	70	
Age [ x− ± s, years]	43.31 ± 13.87	44.17 ± 14.48	0.721
Gender			0.290
Male [*n* (%)]	48 (68.57)	42 (60.00)	
Female [*n* (%)]	22 (31.43)	28 (40.00)	
Mechanism of trauma			0.716
Falling injury [*n* (%)]	21 (30.00)	23 (32.86)	
Car accident injury [*n* (%)]	33 (47.14)	28 (40.00)	
Violent injury [*n* (%)]	5 (7.14)	8 (11.43)	
Sharp object injury [*n* (%)]	2 (2.86)	4 (5.71)	
Other [*n* (%)]	9 (12.86)	7 (10.00)	
Major site of trauma			0.888
Head and neck [*n* (%)]	8 (11.43)	5 (7.14)	
Face [*n* (%)]	4 (5.71)	3 (4.29)	
Thorax [*n* (%)]	13 (18.57)	12 (17.14)	
Abdomen [*n* (%)]	18 (25.71)	15 (21.43)	
Limbs [*n* (%)]	20 (28.57)	25 (35.71)	
Surface [*n* (%)]	1 (1.43)	2 (2.86)	
Multiple areas [*n* (%)]	6 (8.57)	8 (11.43)	
ISS pre-hospitalization score	17.36 ± 13.44	18.49 ± 12.86	0.612
Trauma severity			0.731
ISS score ≤ 16[*n* (%)]	40 (57.14)	42 (60.00)	
ISS score >16[*n* (%)]	30 (42.86)	28 (40.00)	

### 3.2. Consistency and accuracy of the smart bracelet

Patients’ blood pressure, heart rate, blood oxygen saturation, respiratory rate, and temperature were measured *via* a smart bracelet and compared with the same metrics obtained *via* traditional devices. A paired rank-sum test was performed; the result is shown in Table 2. No significant differences were found between groups (*p* > 0.05), which indicates a high consistency between the smart bracelet and traditional methods. Yet, the results of the first measurements for blood pressure (*K* = 0.862), heart rate (*K* = 0.899), blood oxygen saturation (*K* = 0.605), respiratory rate (*K* = 0.751), and temperature (*K* = 0.635) prior to hospitalization measured *via* smart bracelet were more accurate, and these results were considered statistically significant (*p* < 0.001).

**Table 2:** Analysis of consistency and accuracy in initial measurement results between the smart bracelets and traditional devices in the pre-hospital setting.

Project	Traditional device	Smart bracelet	Z/Kendall coefficient	*p*
Differences in initial measurements between both device types				
Systolic blood pressure [Media(IQR),mmHg]	126 (111.5–146.3)	127.5 (111.0–148.3)	−1.704	0.088
Heart rate [Median(IQR),/min]	91 (77.8–106.0)	92 (78.5–104.8)	−0.003	0.997
Blood oxygen saturation [Median(IQR),%]	97 (95.8–98.0)	97 (97.0–98.0)	−1.653	0.098
Respiratory rate [Median(IQR),%]	15 (13.0–19.0)	15 (12.0–19.3)	−1.238	0.216
Temperature [Median(IQR),°C]	36.6 (36.2–36.9)	36.6 (36.5–36.7)	−0.281	0.779
Consistency of initial measurements between both devices in the pre-hospital setting				
Systolic blood pressure [Media(IQR),mmHg]	126 (111.5–146.3)	127.5 (111.0–148.3)	0.862	<0.001
Heart rate [Median(IQR), /min]	91 (77.8–106.0)	92 (78.5–104.8)	0.899	<0.001
Blood oxygen saturation [Median(IQR),%]	97 (95.8–98.0)	97 (97.0–98.0)	0.605	<0.001
Respiratory rate [Median(IQR),%]	15 (13.0–19.0)	15 (12.0–19.3)	0.751	<0.001
Temperature [Median(IQR),°C]	36.6 (36.2–36.9)	36.6 (36.5–36.7)	0.635	<0.001

### 3.3. Comparison of rescue efficiency

The rescue interventions and treatment efficiencies of both patient groups were compared. The results showed that the time to administration of first-aid first life-saving intervention (*t* = 2.040, *p* = 0.049) and blood transfusions (*t* = 2.310, *p* = 0.048), as well as the use of hemostatic drugs (*t* = 4.416, *p* < 0.001) were significantly shorter for patients with smart bracelets compared to the control group (Table 3), thus suggesting that smart bracelets may improve pre-hospital life-saving interventions (*p* < 0.05). However, when the efficiency of pre-hospital life-saving interventions was discussed separately, including tracheal intubation, fluid replenishment, and needle decompression, there was no significant difference between the two groups (all *p* > 0.05). The efficiency of in-hospital life-saving interventions, including blood transfusion (*p* < 0.05) and the use of hemostatic drugs (*p* < 0.05), for patients in the experimental group was better than that of the control group. Furthermore, the time to trauma team engagement for patients with smart bracelets was 3.

**Table 3:** Comparison of rescue efficiencies between groups.

Project	Control group	Test group	χ^2^/t	*p*
Administration of at least one life-saving intervention prior to hospitalization [*n* (%)]	20 (28.57)	18 (25.17)	0.144	0.704
Amount of time before use of first life-saving intervention prior to hospitalization [ x− ± s, Min]	6.65 ± 3.12	4.83 ± 2.24	2.040	0.049
Tracheal intubation prior to hospitalization [n (%)]	6 (8.57)	5 (7.14)	0.099	0.753
Amount of time before intubation prior to hospitalization [ x− ± s, Min]	4.67 ± 3.51	3.68 ± 2.58	0.520	0.616
Fluid replenishment prior to hospitalization [*n* (%)]	16 (22.86)	13 (18.57)	0.391	0.532
Time to initiation of fluid replenishment prior to hospitalization [ x− ± s, Min]	6.97 ± 2.84	5.27 ± 2.03	1.802	0.083
Needle decompression prior to hospitalization [*n* (%)]	2 (2.86)	1 (1.43)		1
Emergency blood transfusion [*n* (%)]	8 (11.43)	6 (8.57)	0.317	0.573
Starting time of blood transfusion [ x− ± s, Min]	163.25 ± 83.44	91.67 ± 23.27	2.310	0.048
Emergency use of hemostatic drugs [*n* (%)]	33 (47.14)	29 (41.43)	0.365	0.546
Duration of emergency hemostatic drug use [ x− ± s, Min]	36.91 ± 7.70	25.62 ± 11.72	4.416	<0.001
The situation of trauma team activation prior to arriving at the hospital				
Start a Trauma Team [*n* (%)]	30 (42.86)	28 (40.00)	0.118	0.731
Time to trauma team activation [ x− ± s, Min]	8.22 ± 3.76	5.80 ± 3.04	2.709	0.009

### 3.4. Comparison of treatment effects

When comparing the treatment effects in both groups of patients, the duration of stay in the emergency room (ER) was significantly shorter for patients wearing the smart bracelet compared to the control group (*t* = 2.075, *p* = 0.043). Furthermore, there were no significant differences in mortality rates between both groups within 24-h post-admission to the ER or on day-28 of patient care (*p* > 0.05) (Table 4).

**Table 4:** Comparison of treatment effects for both groups of patients.

Project	Control group	Test group	*χ*^2^/t	*p*
24-h mortality rate [*n* (%)]	2 (2.86)	0 (0.00)		0.496
28-day mortality rate [*n* (%)]	3 (4.29)	1 (1.43)		0.620
Number of patients needing resuscitation [*n* (%)]	25	20	0.819	0.366
Patient length of stay in the emergency room [ x− ± s, Min]	199.60 ± 71.67	159.36 ± 65.29	2.075	0.043

## 4. Discussion

This study validated the consistency and accuracy of a multi-parameter integrated life monitoring smart bracelet based on WBAN technology for use prior to hospitalization and studied the impact of combined WBAN and remote 5G technology on treatment efficiency and outcomes for these trauma patients.

Compared with traditional equipment, small and integrated monitoring devices benefit medical personnel performing treatments on trauma patients while increasing overall patient compliance (2, 11). Wearable devices have been widely used in healthcare for personalized diagnosis and treatment systems, and their effectiveness has been demonstrated in rehabilitation medicine, intraoperative monitoring, sports medicine, and other fields of research (8, 12, 13). However, the application of a BAN to emergency medical care has not yet been reported. Moreover, the literature on the accuracy and clinical benefits of wearable devices is still limited (14).

The results from this study provide additional information on the accuracy of wearable devices for use in the field of emergency medical care. In this study, we found no significant statistical difference (*p* > 0.05) between blood pressure, heart rate, blood oxygen saturation, respiratory rate, and temperature measurements in the experimental group (with smart bracelet) and control patients (with traditional devices) prior to hospitalization. Yet, the consistency of smart bracelet measurements for blood pressure (*K* = 0.862), heart rate (*K* = 0.899), blood oxygen saturation (*K* = 0.605), respiratory rate (*K* = 0.751), and temperature (*K* = 0.635) was superior compared with the measurements obtained *via* traditional devices (all *p* < 0.001). Although our results suggest that the smart bracelet demonstrates a high degree of accuracy with regard to the measurement of vital signs, measurement errors cannot be ruled out. Yet, to the best of our knowledge, no study has validated the accuracy of wearable devices for use in trauma patients prior to hospitalization.

In China, there is a shortage of the equipment used for emergency care before hospitalization. Therefore, using integrated and portable devices may significantly improve the efficiency of emergency care for these patients. Liu et al. showed that using a portable wireless life monitoring device during trauma care before hospitalization could improve the accuracy of predicting life-saving interventions for patients (15). Furthermore, wearable devices achieve real-time data transmission through wireless and human-computer interaction technology, thus allowing medical staff to remotely and instantaneously understand a patient’s physical condition. Furthermore, high levels of integration and the compactness of wearable devices make them more environmentally friendly (16). The smart bracelet used in our study not only monitors vital signs in real-time during emergencies but can also be used for remote medical assistance through the use of body area networks and remote 5G technology. Our results suggested that the use of a BAN-based smart bracelet in emergency care prior to hospitalization can implement life-saving interventions in a more timely manner compared to conventional emergency care techniques, including first life-saving intervention (*t* = 2.040, *p* = 0.049), blood transfusion (*t* = 2.310, *p* = 0.048) and the use of hemostatic drugs (*t* = 4.416, *p* < 0.001). When multiple life-saving interventions (i.e., tracheal intubation, fluid resuscitation, needle decompression) from our study were separately analyzed, no significant difference was found between the groups. On-site tracheal intubation is a challenging procedure, with questionable short-term benefits. First responders often lack experience in this technique, leading to delayed or repeated intubation, which increases the risk of death (17). Therefore, using efficient and portable devices to shorten on-site assessment time may lead to quicker intubation, fluids and needle decompression administration. However, the small number of patients in our study introduced significant variability in the results, making it impossible to draw a clear conclusion.

Overall, the experimental group received life-saving interventions faster than the control group. Furthermore, we also found that patients in the experimental group received assistance from the trauma teams in a shorter period thanks to the 5G remote medical assistance (*t* = 2.709, *p* = 0.009). Previous studies have shown that timely and effective life-saving interventions can reduce mortality rates among trauma patients and that remote communication with emergency surgeons significantly improves the effect of life-saving interventions as well as reduces overall mortality rates in trauma patients (18, 19). Collaborative treatment between on-site and intra-hospital care can improve the diagnosis efficiency and treatment of severely injured patients (20). It is currently undisputed that minimizing the time from a severe injury to treatment is important; however, our results showed no significant difference in 24-h and 28-day mortality rates between groups. The overall number of patients who died in our study was small, and the results we obtained contained significant variation. Therefore, we could draw no clear conclusion from this data. The smart bracelets group had shorter stays in the emergency department than the control group (*t* = 2.075, *p* = 0.043). This is most likely due to the smart bracelet technology that reduced patient admission time and increased the number of resources available to patients in the emergency department (20, 21). For patients receiving emergency care before hospitalization, BAN can be used to perform simultaneous multi-user monitoring, which is more effective for monitoring the health statuses of patients on-site and coordinating large-scale casualty treatment when necessary (10, 20, 21).

The present study has a few limitations: (1) this is an observational study, and the results are inevitably subject to confounding factors. However, we effectively controlled these factors by using random grouping for the experiment. The general conditions of both patient groups (i.e., age, gender, trauma type, pre-hospitalization ISS score, and trauma severity) were compared, showing no statistical differences. (2) Although we compared the baseline data of the two groups of patients and found no significant statistical difference in the results (Table 1), not all samples were subjected to life-saving interventions (Table 3), and there may be some bias in the baseline data of those who subjected to life-saving interventions between two groups. To some extent, group randomization reduces the possibility of such bias, and further study should have more specific trauma samples or larger samples for stratified analysis. (3) There are differences in clinical experience among different clinical decision-makers, and the difference in enthusiasm for implementing life-saving interventions may have a certain degree of interference with the results, which were not evaluated. (4) In order to identify the advantages of using a BAN-based smart bracelet, future studies should include a separate experimental group that will use this system so as to reduce bias. However, there is currently insufficient evidence to determine whether the results of wearable devices used in pre-hospital settings are reliable. In a major accident, medical staff may be more inclined to focus on traditional equipment during the pre-hospital treatment period for each patient, which we did not evaluate during this study.

## 5. Conclusion

A first aid smart bracelet based on body area network technology can improve the treatment efficiency and effectiveness of trauma care in patients pre-hospitalization. Emergency smart bracelets can shorten the start time of a patient’s first life-saving intervention, such as a blood transfusion, administering hemostatic drugs, and notification of the trauma team, and reduce the time spent in the emergency room. However, the results of this study did not suggest that smart bracelets made a significant difference concerning patient survival. Therefore, we provided an effective technical mean for emergency doctors to improve both efficiency and efficacy of emergency treatment; however, further research and verification are needed.

## Data availability statement

The raw data supporting the conclusions of this article will be made available by the authors, without undue reservation.

## Ethics statement

The studies involving human participants were reviewed and approved by the Ethics Review Committee of the General Hospital of Shenzhen University. The patients/participants provided their written informed consent to participate in this study.

## Author contributions

WH, J-YY, RL, LY, and J-QF completed the data collection and organization. WH, J-YY, H-JF, and S-KH analyzed the data and completed the first draft of the manuscript. All authors contributed to the article and approved the submitted version.

## Funding

This research was supported by Shenzhen Medical and Health Three Project Support (Project no. SZSM201911007); Shenzhen University Stability Support Program, (Project no. 20200824145152001) “5G+ Medical and Health Application Pilot Project” of Ministry of Industry and Information and National Health Commission (Guangdong Province, Direction 1:5G + Emergency Treatment, 11).

## Conflict of interest

The authors declare that the research was conducted in the absence of any commercial or financial relationships that could be construed as a potential conflict of interest.

## Publisher’s note

All claims expressed in this article are solely those of the authors and do not necessarily represent those of their affiliated organizations, or those of the publisher, the editors and the reviewers. Any product that may be evaluated in this article, or claim that may be made by its manufacturer, is not guaranteed or endorsed by the publisher.
